# Supplementary material for: The origin of carbon isotope vital effects in coccolith calcite
Source: Nat Commun. 2017 Mar 6;8:14511. doi: 10.1038/ncomms14511 (PMC5343501; doi:10.1038/ncomms14511)
Supplement: Supplementary Information — Supplementary Note, Supplementary Figures and Supplementary References [file ncomms14511-s1.pdf]

Supplementary note 1

Implications of boundary layer disequilibrium

Although DIC will never be a biomass limiting nutrient for phytoplankton in the ocean, it has been suggested that those relying on the passive diffusive supply of CO_2 alone (in the absence of CA) may be growth rate limited by the rate of CO_2 supply to the surface of the cell (supplementary Fig.3a)[1,2]. If the assimilation rate of carbon by a cell exceeds the reacto-diffusive supply rate, some mechanism of enhancing the supply to the cell must exist. The carbon supply rate to the cell in the absence of such a mechanism is the sum of HCO_3^- conversion to CO_2 within the boundary layer (reactive component), and the diffusion of CO_2 from the bulk medium into the boundary layer (diffusive component). Calculations given in[3] based on a cellular organic carbon density of 20 fmol μm^{-3} (supplementary Fig.3b) suggest that under conditions typical of the modern ocean, cells with a radius of about 10 μm can sustain a maximum division rate of ~1.0 per day (supplementary Fig.3c). This estimate is also based on the assumption that the concentration of CO_2 at the surface of the cell is 1/3rd the concentration of the bulk medium, which is reasonable; complete depletion of CO_2 in the boundary layer is not possible because cells that rely on the diffusive supply of CO_2 alone will leak CO_2 from their cells back into the boundary layer.

The numbers presented by[3] are not quite representative of coccolithophores however. Although the results from the current study show that the cellular organic carbon density is very close to 20 fmolμm^{-3} in the species studied here (supplementary Fig.3b), calcification also occurs intracellularly, so carbon used for calcification is additionally taken up across the same cellular membrane. The total cellular carbon density (organic plus inorganic carbon per unit cellular volume) in coccolithophores is ~43 fmolμm^{-3} (supplementary Fig.3b), which is approximately double that of non-calcifiers. Additionally, the vast majority of calcification and photosynthesis in coccolithophores occurs in the light[4], which means that the instantaneous carbon assimilation rate (which is relevant for comparing with the reacto-diffusive supply rate of CO_2) is approximately double that estimated through the cellular carbon content and division rate. Given these assumptions, the maximum division rate of a coccolithophore cell with a radius of 10 μm in the modern ocean would be more like 0.25 per day - a quarter of that predicted by[3] (supplementary Fig.3d).

In the experiments conducted in this study, no carbon assimilation rates exceeded the maximum reacto-diffusive supply rate of CO_2, assuming that the minimum CO_2 concentration at the surface of the cell could be as low as 1/3rd that of the bulk medium (supplementary Fig.3e). The actual theoretical concentration of CO_2 at the surface of the cell, calculated from the observed assimilation rate, can however become highly depleted, reaching values as low as 50% that of the bulk medium (supplementary Fig.3f). Depletion such as this has implications for the rate of diffusion of CO_2 across the cellular membrane, and therefore for proxies such as the Alkenone CO_2 proxy, which assumes that DIC is supplied to the cell solely as a passive-diffusive supply of CO_2.

In this model, it is explicitly assumed that CA_e is present and active in all species, and that the concentration of CO_2 at the cell's surface can be assumed to equal that of the bulk medium. This is most likely true for *E. huxleyi*[5–7], but is less clear for other species such as *G. oceanica* and *C. pelagicus*[8]. In these other species, it may therefore be possible that the microenvironment at the surface of the cell is depleted in CO_2, which would simply be manifest as a greater sensitivity to [CO_2].

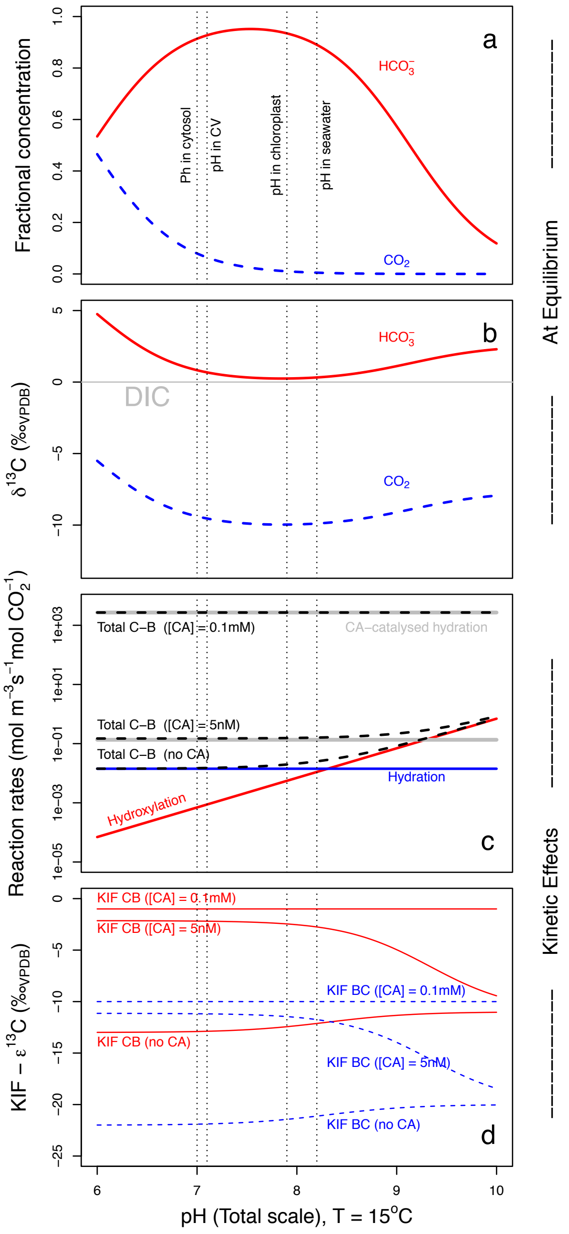

Supplementary Figure 1. Carbonate chemistry and isotopes, Effects of pH on: **a:** Equilibrium concentrations of CO_2 and HCO_3^- (as a fraction of total DIC), **b:** Equilibrium isotopic compositions of CO_2 and HCO_3^- (Relative to DIC), **c:** Reaction rates within DIC system. Hydration, hydroxylation and CA-catalysed hydration in blue (dashed), red and grey respectively, and total rate of conversion of CO_2 to HCO_3^- in black (dashed), at different CA concentrations. **d:** Net pH-dependent kinetic fractionation factors for conversion of CO_2 to HCO_3^- (CB; red) and of HCO_3^- to CO_2 (BC; blue, dashed), at the same CA concentrations as (C). Plotted for context,[9] deduced intracellular CA activities which correspond to a concentration of ~ 0.1 mM assuming a specific activity of CA of 2.7×10^7 s^{-1} [10]. Hydration and hydroxylation reactions are unimportant when CA concentrations are greater than ~ 1 μM. Above this, as long as the substrate concentration is well below the half saturation concentration of CA, the rate of CA-catalysed hydration/dehydration scales linearly with the concentration of CA (see methods).

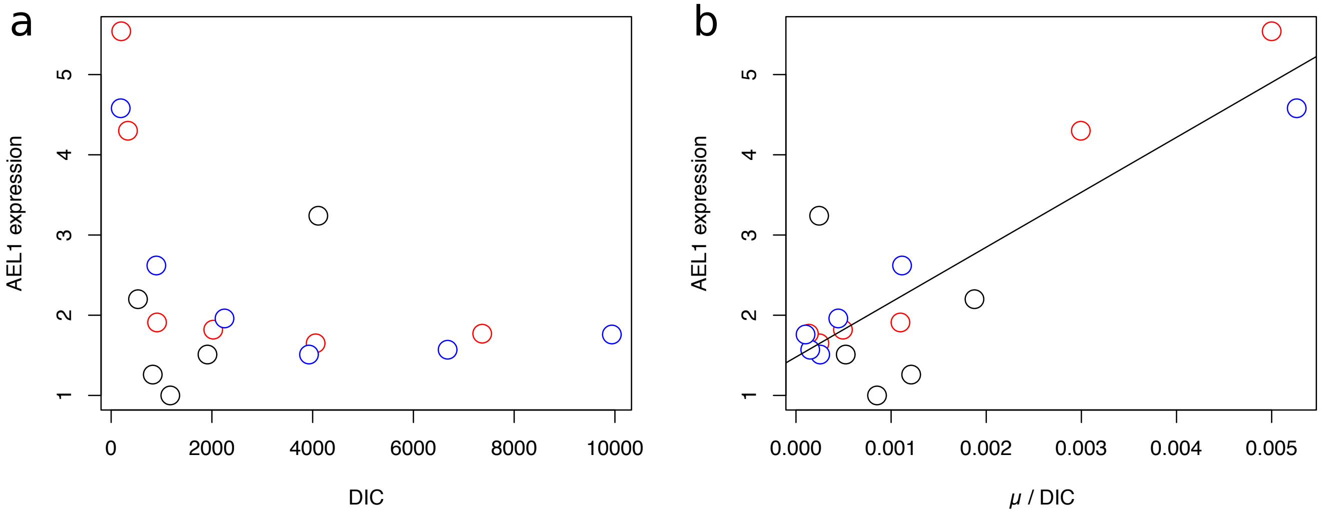

Supplementary Figure 2. Interpretation of the data of Bach et al. 2013[5], a: Transcript abundance of the AEL1 protein against [DIC]. **b:** As left but plotted against (growth rate / [DIC]) which at constant cell size, carbon density and membrane permeability is approximately proportional to utilization.

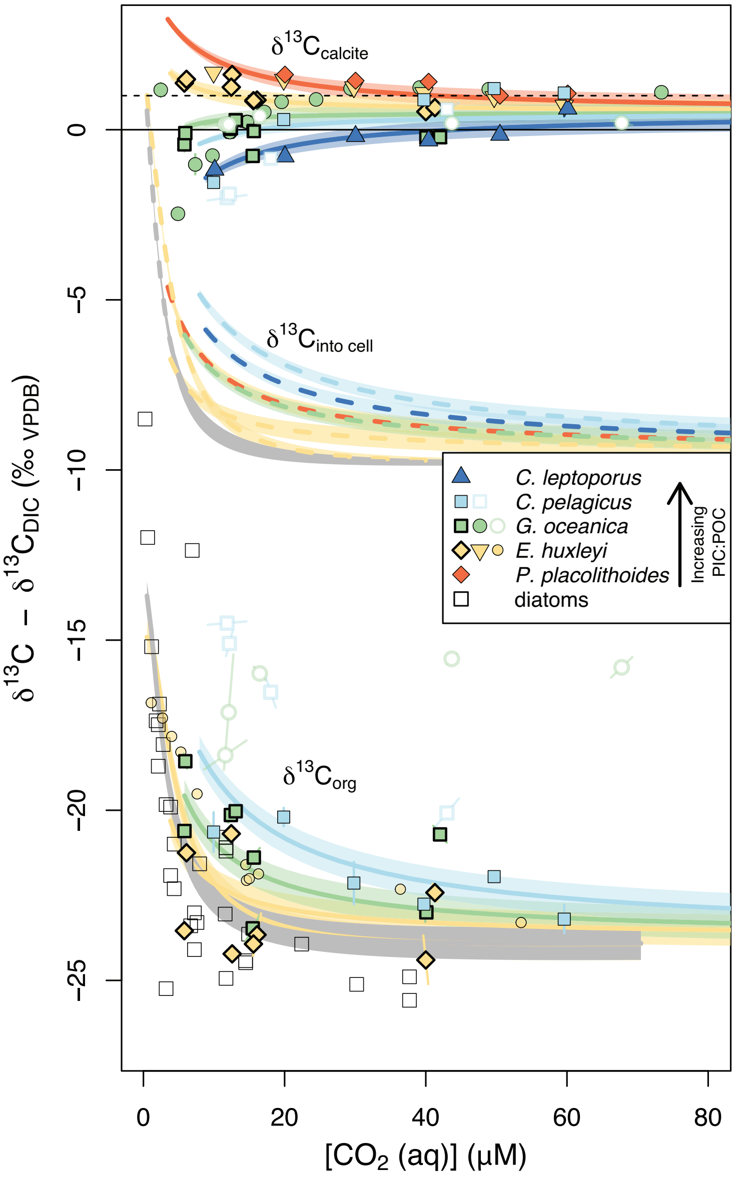

Supplementary Figure 3. Model output, Model output across the experimental range of CO_2, with empirical data superimposed. In addition to the data shown in Fig. 2 are data from dulite batch cultures of diatoms[11–13] compiled by Riebesell et al. (2000). For completion, also included are data from Rickaby et al. (2010)[14]. Although the calcite carbon isotopic compositions overlay the trends in the other data presented here, the organic carbon isotopes are questionable, particularly those of *G. oceanica* which are inconsistent with all other datasets in the literature, which unanimously describe an increasing magnitude of isotopic fractionation of carbon into organic matter with increasing CO_2 concentrations. The parameter values held constant across all values of CO_2 used to generate the representative model curves (cell radius, division rate, PIC:POC) are respectively as follows: *E. huxleyi* (2.3 μm, 1 day^{-1}, 0.5), *G. oceanica* (3.5 μm, 1 day^{-1}, 1.1), *C. pelagicus* (8 μm, 0.9 day^{-1}, 1.2), *C. leptoporus* (6 μm, 0.65 day^{-1}, 2.2), *P. placolithoides* (7 μm, 0.8 day^{-1}, 0.3) and the diatom *Phaeodactylum tricornutum* (2 μm, 1 day^{-1}, 0). These values for each species are representative only, and are taken from across a range of sources in the literature and from our own unpublished data. Note: The carbonate chemistry of the dilute batch experimental data for diatoms, and for the *E.huxleyi* data of Riebesell et al. (2000)[15], was manipulated with acid addition rather than DIC manipulation, and the model input was altered to reflect this. The output is an enhancement of HCO_3^- uptake at low CO_2.

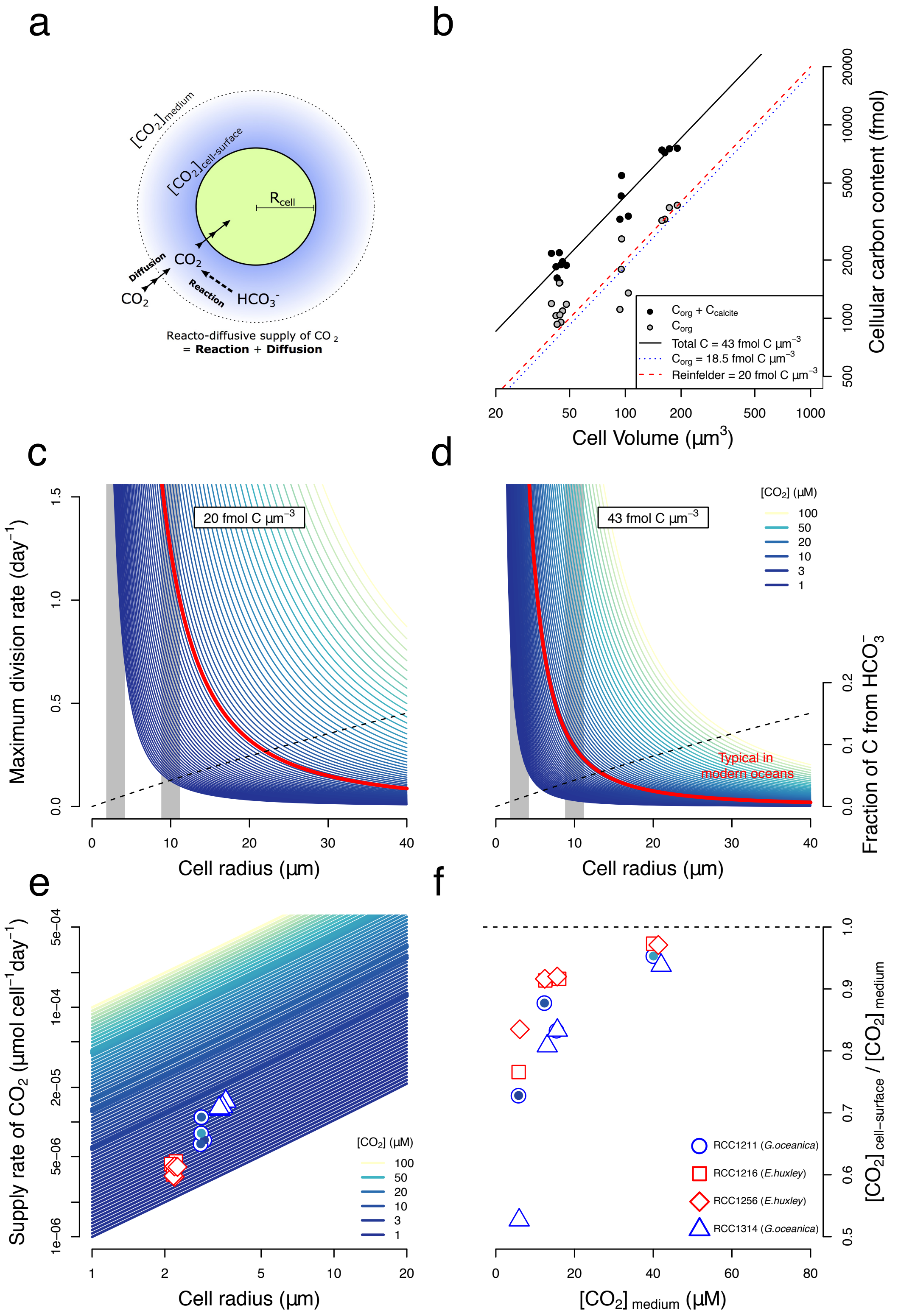

Supplementary Figure 4. Considerations of the diffusive boundary layer around the cell. a: Schematic representation of cellular boundary layer, **b:** Estimates of organic and total carbon density per unit cellular volume, **c & d:** Maximum possible division rate sustainable given different assumed cellular carbon densities, **e:** Carbon usage by coccolithophores in each experiment; fill of points corresponds to experimental [DIC] condition. The four thick lines correspond to the four experiments, **f:** Theoretical $[CO_2]$ depletion at the surface of coccolithophores in each experiment. See supplementary note 1 for details.

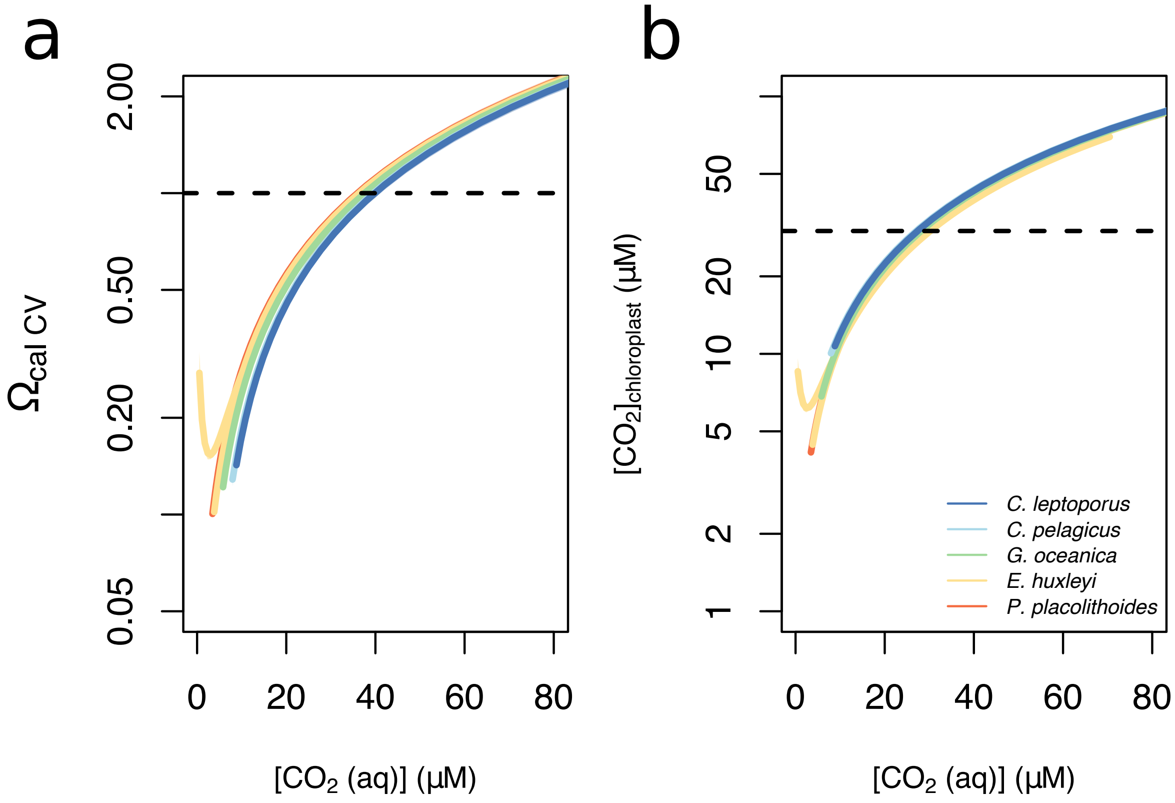

Supplementary Figure 5. **$\Omega_{cal\ CV}$ and $[CO_2]_{chloroplast}$ based on model output, with pH values of Anning et al. (1996)**[16]. **a:** Calcite saturation state in the coccolith vesicle. Dashed line highlights the value of Ω_{cal} = 1, below which calcite is thermodynamically unstable. $\Omega_{cal} = [Ca^{2+}][CO_3^{2-}] / K_{sp}$, where K_{sp} is a strong function of salinity[17]. $[CO_3^{2-}]$ is inferred from ph_{CV} and $[HCO_3^-]$ assuming chemical equilibrium between $HCO_3^-$and CO_3^{2-}. As these two chemical species rapidly reach chemical equilibrium, at the calcification rates considered here this assumpton is justified[18]. The value pf K_{sp} used here is 3.7×10^{-9}, and $[Ca^{2+}]$ in the coccolith vesicle was assumed to be 500μM[19]. **b:** Concentration of carbon dioxide in the chloroplast. The dashed line represents a typical estimated half saturation constant (K_m) of RuBisCO in *E. huxleyi*[20]. This is a rough measure of the concentration of CO_2 at which RuBisCO operates most efficiently. Below this value, RuBisCO operates at sub-optimal velocities, and above this value, the enzymatic saturation begins to become apparent.

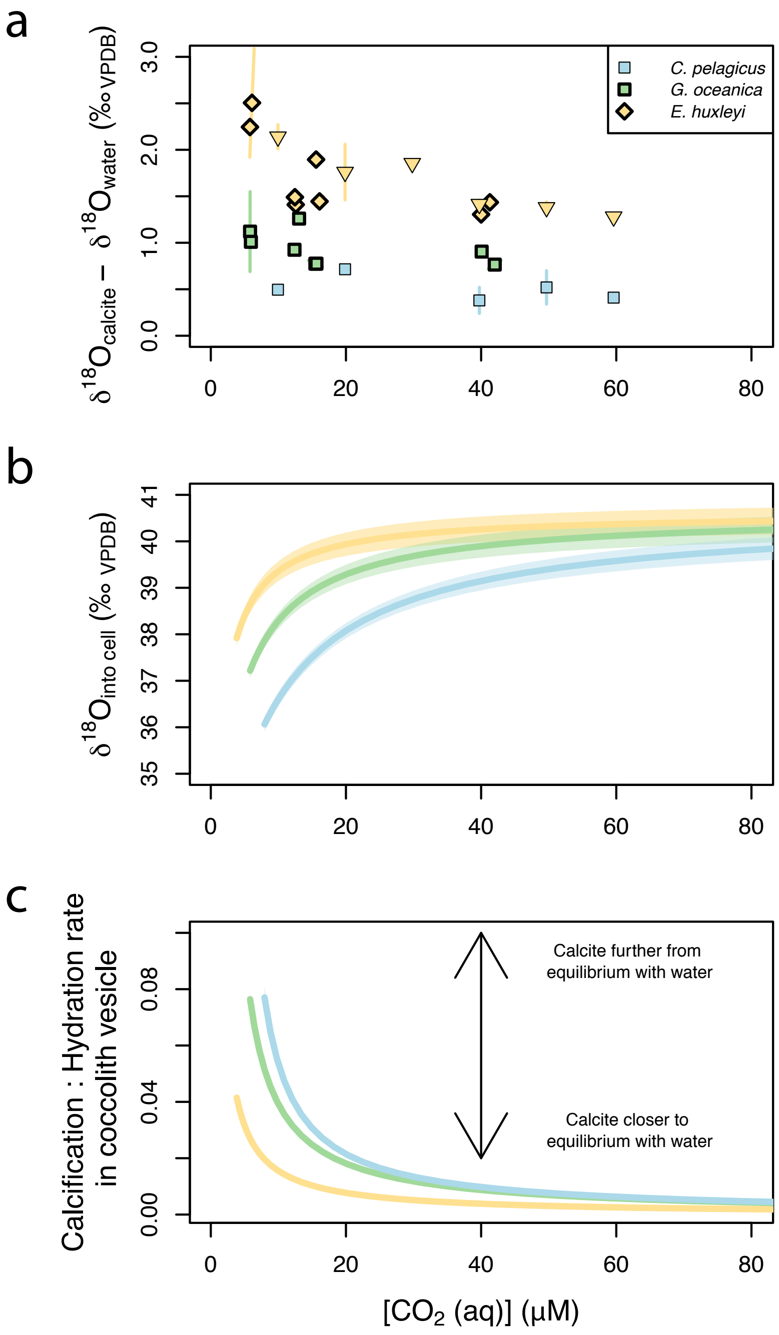

Supplementary Figure 6. Data and model output on which discussion of oxygen isotopes is based. **a:** Oxygen isotopic compositions of calcite relative to DIC, **b:** Model output describing the oxygen isotopic composition of all carbon entering the cell (i.e. in the form of CO_2 and HCO_3^-), **c:** Ratio of the rates of calcification to hydration in the coccolith vesicle. This ratio is a measure of the number of hydration/dehydration cycles each carbon atom has undergone. The higher this ratio, the further from equilibrium the DIC pool. Note how oxygen isotopic vital effects appear to converge at high CO_2 (top), which is consistent with oxygen in the carbonate system being further towards equilibrium with water.

Supplementary References

[1] Riebesell, U., Wolf-Gladrow, D. A. & Smetacek, V. Carbon dioxide limitation of marine phytoplankton growth rates. *Nature* **361**, 249–251 (1993). DOI 10.1038/361249a0.

[2] Gavis, J. & Ferguson, J. Kinetics of carbon dioxide uptake by phytoplankton at high pH1. *Limnology and Oceanography* **20**, 211–221 (1975).

[3] Reinfelder, J. R. Carbon concentrating mechanisms in eukaryotic marine phytoplankton. *Annual review of marine science* **3**, 291–315 (2011). DOI 10.1146/annurev-marine-120709-142720.

[4] Müller, M., Antia, A. & LaRoche, J. Influence of cell cycle phase on calcification in the coccolithophore Emiliania huxleyi. *Limnology and Oceanography* **53**, 506–512 (2008).

[5] Bach, L. T. *et al.* Dissecting the impact of CO2 and pH on the mechanisms of photosynthesis and calcification in the coccolithophore Emiliania huxleyi. *New Phytologist* **199**, 121–134 (2013). DOI 10.1111/nph.12225.

[6] Richier, S., Fiorini, S., Kerros, M.-E., von Dassow, P. & Gattuso, J.-P. Response of the calcifying coccolithophore Emiliania huxleyi to low pH/high pCO2: from physiology to molecular level. *Marine biology* **158**, 551–560 (2011). DOI 10.1007/s00227-010-1580-8.

[7] Richier, S. *et al.* Light-dependent transcriptional regulation of genes of biogeochemical interest in the diploid and haploid life cycle stages of Emiliania huxleyi. *Applied and environmental microbiology* **75**, 3366–9 (2009). DOI 10.1128/AEM.02737-08.

[8] Nimer, N. a., IglesiasRodriguez, M. D. & Merrett, M. J. Bicarbonate utilization by marine phytoplankton species. *Journal of Phycology* **33**, 625–631 (1997). DOI 10.1111/j.0022-3646.1997.00625.x.

[9] Hopkinson, B. M., Dupont, C. L., Allen, A. E. & Morel, F. M. M. Efficiency of the CO2-concentrating mechanism of diatoms. *Proceedings of the National Academy of Sciences of the United States of America* **108**, 3830–7 (2011). DOI 10.1073/pnas.1018062108.

[10] Uchikawa, J. & Zeebe, R. The effect of carbonic anhydrase on the kinetics and equilibrium of the oxygen isotope exchange in the CO 2ÃćÂĂÂŞH 2 O system: Implications for δ 18 O vital effects in. *Geochimica et Cosmochimica Acta* **95**, 15–34 (2012). DOI 10.1016/j.gca.2012.07.022.

[11] Riebesell, U., Burkhardt, S., Dauelsberg, A. & Kroon, B. Carbon isotope fractionation by a marine diatom: Dependence on the growth-rate-limiting resource. *Marine Ecology Progress Series* **193**, 295–303 (2000). DOI 10.3354/meps193295.

[12] Burkhardt, S., Riebesell, U. & Zondervan, I. Stable carbon isotope fractionation by marine phytoplankton in response to daylength, growth rate, and CO2 availability. *Marine Ecology Progress Series* **184**, 31–41 (1999). DOI 10.3354/meps184031.

[13] Johnston, A. M. The effect of environmental variables on 13C discrimination by two marine phytoplankton. *Marine Ecology Progress Series* **132**, 257–263 (1996). DOI 10.3354/meps132257.

[14] Rickaby, R. E. M., Henderiks, J. & Young, J. N. Perturbing phytoplankton: response and isotopic fractionation with changing carbonate chemistry in two coccolithophore species. *Climate of the Past* **6**, 771–785 (2010). DOI 10.5194/cp-6-771-2010.

[15] Riebesell, U., Revill, A. T., Holdsworth, D. G. & Volkman, J. K. The effects of varying CO2 concentration on lipid composition and carbon isotope fractionation in Emiliania huxleyi. *Geochimica et Cosmochimica Acta* **64**, 4179–4192 (2000). DOI 10.1016/S0016-7037(00)00474-9.

[16] Anning, T., Nimer, N., Merrett, M. J. & Brownlee, C. Costs and benefits of calcification in coccolithophorids. *Journal of Marine Systems* **9**, 45–56 (1996). DOI 10.1016/0924-7963(96)00015-2.

[17] Mucci, A. The Solubility of Calcite and Aragonite in Sea Water at Various Salinities, Temperatures, and one atmospheric total pressure. *American Journal of Science* **283**, 780–799 (1983). DOI 10.2475/ajs.283.7.780.

[18] Zeebe, R. & Wolf-Gladrow, D. *CO2 in Seawater: Equilibrium, Kinetics, Isotopes* (Elsevier, 2001).

[19] Langer, G. *et al.* Coccolith strontium to calcium ratios in Emiliania huxleyi: The dependence on seawater strontium and calcium concentrations. *Limnology and Oceanography* **51**, 310–320 (2006). DOI 10.4319/lo.2006.51.1.0310.

[20] Badger, M. & Andrews, T. The diversity and coevolution of Rubisco, plastids, pyrenoids, and chloroplast-based CO2-concentrating mechanisms in algae. *Canadian Journal of Botany* **1071**, 1052–1071 (1998).
